# Thirty years since the great vascular revolution: the birth of a new era

**DOI:** 10.1590/1677-5449.200192

**Published:** 2021-03-24

**Authors:** Tulio Pinho Navarro, Ricardo Jayme Procópio

**Affiliations:** 1 Universidade Federal de Minas Gerais – UFMG, Belo Horizonte, MG, Brasil.; 2 Universidade Federal de Minas Gerais – UFMG, Hospital das Clínicas, Belo Horizonte, MG, Brasil.

At the end of August, in 1990, Dr. Juan Carlos Parodi answered a telephone call from the president of the Republic of Argentina, Carlos Menen. The president asked him to assess his 70-year-old cousin, a smoker with severe chronic obstructive pulmonary disease, who had an abdominal aortic aneurysm with a diameter of 6 cm and had recently been suffering from severe lumbar pain. There was an imminent risk of rupture. The assisting doctors had said that he would not survive surgery, due to his severe lung disease.^1^

Dr. Parodi had been trained in his specialty at the famous Cleveland Clinic, in the United States, in the mid-1970s. When he returned to Argentina, Dr. Parodi started to research an idea he had had when abroad, specifically aimed at treating high-risk patients who had elevated mortality, even at what was then the largest surgical center in the world. He started to conduct experimental studies on dogs in 1976, constructing aneurysms in canine aortas with aneurysm-shaped polyester grafts (Dacron).^1^

In parallel, another Argentinean surgeon, Dr. Julio Palmaz, had studied medicine in La Plata and then trained in Vascular Radiology, moving to the United States in 1977 and becoming Head of Angiography and Special Procedures at the Radiology Department of the University of Texas Health Science Center at San Antonio (UTHSCSA) in 1983. After attending a lecture by Andreas Grutzing (inventor of the angioplasty balloon, based on the balloon catheter created by Thomas Fogarty) in 1978, Palmaz had the idea of fitting a metal mold to the balloon, inventing the stent. Julio Palmaz registered the patent in 1985, and the devices were marketed by Johnson & Johnson.

Parodi had the idea of combining the stent created by Julio Palmaz with a conventional polyester vascular graft using a suture at the upper extremity of the stent. This combined device was placed in a 22F Teflon introducer.^1^

Carlos Menen, aware of Parodi’s work, asked him for his opinion on the case of his cousin, described above. Parodi explained to the president and the patient that his alternative technique had only ever been used in dogs, never in a human being. After considering the possibilities, the patient and the president agreed to the attempt and a consent form was signed.

On the morning of Thursday, September 6, 1990, at the Instituto Cardiovascular of Buenos Aires, Dr. Parodi, with his colleagues Julio Palmaz and Hector Barone, performed the procedure under epidural anesthesia, making just a single incision in the right groin to access the common femoral artery.^1^ The procedure was a success, with total exclusion of the aneurysm. The patient underwent angiography at 53rd postoperative day, which demonstrated maintenance of total aneurysm exclusion. The patient survived for another 9 years, before dying due to pancreatic cancer.^1^

On the same day, September 6, 1990, they operated on a second case, a 68-year-old woman. However, they failed to identify the proximal marking on the stent and the endoprosthesis was inadvertently released 3 cm below the planned position and the polyester graft completely entered the right iliac artery, occluding the left iliac artery. It was necessary to perform a laparotomy and convert to conventional surgery. When the aorta was opened, the team found that the stent was right against the wall of the vessel. In the end, the patient tolerated the procedure well and was discharged from hospital a few days later.^1^

On November 11, 1990, a 63-year-old patient who had had a stroke a few days earlier began to complain of intense abdominal pains and was diagnosed with an acute dissection of the abdominal aorta with formation of an aneurysm. The same day, the patient underwent endoprosthesis implantation, with proximal infrarenal sealing of the entry site, without deployment of a distal stent. The pain receded during the postoperative period. At 7-month follow-up, the diameter of the aneurysm had reduced and the false lumen was occluded.^1^

On January 3, 1991, another 61-year-old patient, with an aneurysm with a 6.5 cm diameter, although asymptomatic, volunteered to undergo the treatment, which was performed successfully the same day. Follow-up at 6 months showed total exclusion of the aneurysm.^1^

On May 26, 1991, a 62-year-old patient who had presented with acute arterial ischemia due to embolization by thrombi of a 3.5 cm abdominal aneurysm also underwent successful exclusion of the aneurysm. After 3 months’ follow-up, abdominal duplex ultrasonography showed total exclusion of the aneurysm, with no further episodes of embolization.^1^

Parodi was certain that a revolution was underway. Confident, he compiled his data and presented his findings to a congress in the United States, in 1991, where he met several of his former colleagues. However, the reaction to the innovation what not as he had expected. The Vascular Establishment reacted very negatively, asking him why he wanted to change a treatment that had become so well-established since the time of Dubost, and later improved by Michael DeBakey, among others. They barely listened to him. All of the major medical journals refused to accept his paper.

While he was attempting to present his new ideas, one participant was observing him closely. He approached Dr. Parodi and said he would like to learn more about his work. This was Dr. John J. Bergan, who died in 2014, a pioneer of kidney transplantation and one of the of those responsible for creating the specialty of Vascular Surgery. Dr. Bergan understood the new concept and saw that Parodi’s proposal could help critical patients, at high risk from surgery. Since the editors of the major journals had refused to publish Parodi’s study, Dr. Bergan contacted a relative, Dr. Ramon Buerger, Editor in Chief of the Annals of Vascular Surgery, a journal with a lower impact factor.

In November 1991, “Transfemoral intraluminal graft implantation for abdominal aortic aneurysms”, by Juan Parodi, Julio Palmaz, and Hector Barone, was published in the Annals of Vascular Surgery.^1^ Publication of this article is a landmark in vascular surgery. The consequence was like a silent bomb, with delayed effects. The vascular world was shocked, but the Vascular Establishment remained opposed.

In August of 1992, a 76-year-old man with oxygen-dependent chronic obstructive pulmonary disease, coronary artery disease, and recurrent ventricular tachyarrhythmia presented at the Montefiore Hospital, in The Bronx, New York with an aneurysm of the infrarenal aorta measuring 7.5 cm in diameter and painful and sensitive to palpation.^2^ Dr. Michael Marin was called to assess the patient and agreed with all of the medical consultants that the risk of any type of standard open surgery was too high. Dr. Marin then called Dr. Frank Veith to discuss the possibility of a repair using an endovascular graft, which was promptly accepted as the only option. It was agreed that both surgeons would travel to Buenos Aires to learn the technique from Dr. Parodi. However, Dr. Parodi said that he didn’t have any cases scheduled at the time. On the other hand, he was going to deliver a lecture to a meeting of interventional cardiologists in Milwaukee, in the United States. He suggested that Drs. Veith and Marin meet with him to discuss the patient and how the treatment with an endovascular graft could be accomplished. Dr. Marin met Dr. Parodi in Milwaukee with the patient’s test results and they agreed that he was a good candidate for endovascular repair. It was agreed that it would be better to treat the patient in New York and that this could be done at the same time as the Montefiore annual meeting, to be held in November 1992.^2^

However, there were several problems to overcome. The first was that Johnson & Johnson, which had acquired the rights to Parodi’s and Palmaz’s patents, needed to approve use of a large Palmaz stent in the United States, since this investigational device had not been approved by the Food and Drug Administration (FDA). Dr. Veith then called Paul Marshall, product director at Johnson & Johnson Interventional Systems (J & JIS) to obtain permission. He was reticent to allow Drs. Parodi, Veith, and Marin to use the stent, fearing that this could compromise the FDA’s assessment of the Palmaz-Schatz coronary stents that were in the final phase of approval.^2^

Veith then requested a meeting with Marvin Woodall, president of J & JIS, to see whether he could be persuaded to allow the procedure on compassionate grounds. Marshall, Woodall, Veith, and Marin met at a restaurant in the Marriott Hotel next to Newark airport. After a 4-hour conversation and expressions of concern for the wellbeing of the patient if he was not treated, the representatives of J & JIS ceded and gave their permission.^2^

When the subject is hospitals in the United States, one thinks of modern installations with a wide range of equipment available. However, although the Montefiore is affiliated to the Albert Einstein College of Medicine, it is located in The Bronx, which is a poor region of New York. The hospital’s installations at the time were not therefore the most modern or best equipped. The Argentinean team were not sure that the procedure would be feasible under those conditions.

Nevertheless, the procedure was performed under local anesthesia on November 23, 1992. Drs. Juan Parodi and Carlos Schonholz, together with Drs. Marin, Veith, and Cynamon, conducted the procedure via a right femoral arteriotomy.^2^ Using an old digital fluoroscopy machine, the graft was positioned and released in the aorta.^2^

The patient’s recovery contrasted favorably with conventional surgery.^2^ Postoperative computed tomography and duplex ultrasonography showed that the aneurysm had been excluded. The patient was discharged a few days later and remained free from symptoms until he succumbed to cardiopulmonary comorbidities, approximately 9 months after the procedure.^2^

Many years before this technique surpassed conventional surgery, Dr. John J. Bergan wrote: “In vascular surgery, no change for the better has occurred that wise and good men have not opposed. Such change is inevitable.”^2^ After this procedure was performed in the United States, the technique spread throughout the world. Dr. Parodi and his team were invited to travel around the whole world to teach and demonstrate this enormous paradigm shift in the vascular universe. The reticent Vascular Establishment gave way. A new era was inaugurated and the vascular universe was never the same again.
