# Supplementary material for: Thirty years since the great vascular revolution: the birth of a new era
Source: J Vasc Bras. 2021 Mar 24;20:e20200192. doi: 10.1590/1677-5449.200192 (PMC8147878; doi:10.1590/1677-5449.200192)
Supplement: Translation

# Translation: Os 30 anos da grande revolução vascular: o nascimento de uma nova era

**Authors:** Tulio Pinho Navarro, Ricardo Jayme Procópio

**Affiliations:** 1 Universidade Federal de Minas Gerais – UFMG, Belo Horizonte, MG, Brasil.; 2 Universidade Federal de Minas Gerais – UFMG, Hospital das Clínicas, Belo Horizonte, MG, Brasil.

O estudo foi realizado no Hospital das Clínicas, Universidade Federal de Minas Gerais (UFMG), Belo Horizonte, MG, Brasil.

No final do mês de agosto de 1990, o Dr. Juan Carlos Parodi recebeu um telefonema do presidente da República Argentina, Sr. Carlos Menen. O presidente solicitou que o médico avaliasse o seu primo tabagista, de 70 anos de idade, com doença pulmonar obstrutiva crônica acentuada, que apresentava um aneurisma de aorta abdominal com 6 cm de diâmetro e estava cursando com dor lombar significativa recente. O risco de ruptura era iminente. Os médicos que o assistiam disseram que não suportaria a cirurgia, uma vez que apresentava doença pulmonar grave^1^.

Dr. Parodi havia feito seu treinamento especializado na renomada *Cleveland Clinic*, nos Estados Unidos, em meados da década de 1970. Quando retornou à Argentina, Dr. Parodi iniciou pesquisas sobre uma ideia que surgiu durante a sua estadia no exterior, justamente para tratar pacientes de alto risco, os quais apresentavam mortalidade elevada, mesmo no então maior centro de cirurgia mundial. Passou a realizar estudos experimentais em cães desde 1976, criando aneurismas em aortas caninas com prótese de poliéster (Dacron) em formato de aneurisma^1^.

Em paralelo, Julio Palmaz, outro cirurgião argentino, após se formar na cidade de La Plata e realizar treinamento em Radiologia Vascular foi para os Estados Unidos em 1977 e posteriormente se tornou Chefe de Angiografia e Procedimentos Especiais no departamento de Radiologia do Centro de Saúde e Ciência da Universidade do Texas em San Antonio (UTHSCSA) em 1983. Após assistir a uma palestra de Andreas Grutzing (inventor do balão de angioplastia, baseado no cateter balão de Thomas Fogarty) em 1978, teve a ideia de colocar um molde de metal no balão e criou o stent. Julio Palmaz recebeu a patente em 1985, cuja comercialização foi feita pela Johnson & Johnson.

Parodi teve a ideia de unir o stent de Julio Palmaz ao enxerto vascular convencional de poliéster por meio de uma sutura do stent na extremidade superior. Esse conjunto era colocado em um introdutor de Teflon de 22F^1^.

Carlos Menen, conhecedor do trabalho de Parodi, solicitou a opinião do médico quanto ao caso descrito anteriormente. Parodi explicou ao presidente e ao paciente que até então a sua técnica alternativa havia sido realizada somente em cães, nunca em um ser humano. Diante das possibilidades, paciente e presidente concordaram com a tentativa e assinou-se o termo de consentimento.

No dia 6 de setembro de 1990, em uma quinta-feira pela manhã, no Instituto Cardiovascular de Buenos Aires, o Dr. Parodi realizou o procedimento sob anestesia epidural, com apenas uma incisão na virilha direita, para acessar a artéria femoral comum, juntamente com seus colegas Julio Palmaz e Hector Barone^1^. O procedimento foi um sucesso, com exclusão completa do aneurisma. Realizou-se ainda uma angiografia no paciente com 53 dias de pós-operatório, tendo demonstrado a manutenção da exclusão completa do aneurisma. O paciente sobreviveu por mais 9 anos, vindo a falecer de câncer pancreático^1^.

Nesse mesmo dia, 6 de setembro de 1990, realizou um segundo caso em uma mulher de 68 anos. Entretanto, houve uma falha em identificar a marca proximal do stent e inadvertidamente a endoprótese foi liberada 3 cm abaixo do planejado e a prótese de poliéster entrou totalmente na artéria ilíaca direita, com oclusão da ilíaca esquerda. Foi necessário realizar uma laparotomia e proceder a cirurgia convencional. Constatou-se, durante a abertura da aorta, que o stent estava bem apropinquado à parede do vaso. Por fim, a paciente tolerou bem o procedimento e recebeu alta hospitalar após alguns dias^1^.

No dia 11 de novembro de 1990, um paciente de 63 anos, após apresentar um acidente vascular encefálico há alguns dias, evoluiu com dor abdominal intensa, tendo sido diagnosticada uma dissecção aguda de aorta abdominal com formação aneurismática. Nesse dia, o paciente foi submetido ao implante da endoprótese com selamento proximal infrarrenal do sítio de entrada, sem utilização de stent distal. A dor cedeu no pós-operatório. No acompanhamento após 7 meses, evidenciou-se redução do diâmetro do aneurisma e oclusão da falsa luz^1^.

No dia 3 de janeiro de 1991, outro paciente de 61 anos com aneurisma de 6,5 cm de diâmetro, porém assintomático, voluntariou-se para submeter-se ao tratamento, que foi realizado nessa data com sucesso. O acompanhamento após 6 meses demonstrou a exclusão completa do aneurisma^1^.

Já em 26 de maio de 1991, um paciente de 62 anos que havia apresentado isquemia arterial aguda por embolização de trombos de um aneurisma abdominal de 3,5 cm foi também submetido com sucesso à exclusão do aneurisma. Após 3 meses de seguimento, o eco-Doppler abdominal mostrou exclusão completa do aneurisma, sem novos episódios de embolização^1^.

Parodi tinha certeza de que uma revolução havia começado. Confiante, juntou seus dados e apresentou em um congresso nos Estados Unidos no mesmo ano de 1991, onde encontrou vários de seus antigos colegas. Entretanto, a reação a essa novidade não foi a antecipada por ele. O *Vascular Establishment* reagiu muito negativamente, tendo questionado a ele por que querer alterar um tratamento tão bem estabelecido desde os tempos de Dubost e aperfeiçoado por Michael DeBakey, entre outros. Mal o ouviram. Todas as grandes revistas médicas se recusaram a receber seu trabalho.

Durante a tentativa de expor suas novas ideias, um participante o observava atentamente. Ele se aproximou do Dr. Parodi e disse-lhe que gostaria de conhecer melhor seu trabalho. Tratava-se do Dr. John J. Bergan, falecido em 2014, pioneiro do transplante renal e um dos responsáveis pela criação da especialidade de Cirurgia Vascular. Dr. Bergan entendeu o novo conceito e que a proposta de Parodi poderia ajudar pacientes graves, com alto risco operatório. Como os editores das maiores revistas se recusaram a publicar o trabalho de Parodi, Dr. Bergan contatou um parente, Dr. Ramon Buerger, Editor-Chefe da revista *Annals of Vascular Surgery*, revista de menor fator de impacto.

Em novembro de 1991, foi publicado o artigo *Transfemoral intraluminal graft implantation for abdominal aortic aneurysms*, de autoria de Juan Parodi, Julio Palmaz e Hector Barone, na revista *Annals of Vascular Surgery* 1. Essa publicação representa um marco na cirurgia vascular. A consequência foi análoga à de uma bomba de efeito silencioso e retardado. O mundo vascular ficou estremecido, porém o *Vascular Establishment* prosseguia contra.

Em agosto de 1992, um homem de 76 anos com doença pulmonar obstrutiva crônica dependente de oxigênio, doença arterial coronariana e taquiarritmia ventricular recorrente apresentou-se com aneurisma de aorta infrarrenal de 7,5 cm de diâmetro, doloroso e sensível a palpação no Hospital Montefiore, Bronx, em Nova York^2^. O Dr. Michael Marin foi solicitado a atender o paciente e concordou com todos os consultores médicos quanto ao risco muito alto para qualquer forma de reparo cirúrgico aberto padrão. O Dr. Marin, então, ligou para o Dr. Frank Veith para discutir a possibilidade de um reparo com enxerto endovascular, que foi prontamente aceita como única opção. Ficou combinado que ambos os cirurgiões viajariam a Buenos Aires para aprender a técnica com Dr. Parodi. Entretanto, Dr. Parodi disse que naquele momento não havia casos agendados. Por outro lado, ele faria uma palestra em um encontro de cardiologistas intervencionistas em Milwaukee, nos Estados Unidos. Ele sugeriu que os Drs. Veith e Marin o encontrassem para discutir sobre o paciente e como o tratamento com enxerto endovascular poderia ser realizado. O Dr. Marin encontrou-se com Dr. Parodi em Milwaukee com os exames do paciente e concordaram que era um bom candidato para o reparo endovascular. Ficou acertado que seria melhor tratar o paciente em Nova York e que isso poderia ser realizado na época da reunião anual do Montefiore, a ser realizada em novembro de 1992^2^.

No entanto, vários problemas tiveram que ser superados. O primeiro era que a Johnson & Johnson, que havia adquirido os direitos das patentes de Parodi e Palmaz, teria que aprovar o uso de um grande stent Palmaz nos Estados Unidos, uma vez que esse dispositivo investigacional não possuía liberação da *Food and Drug Administration* (FDA). O Dr. Veith, então, ligou para Paul Marshall, diretor de novos produtos da Johnson & Johnson Interventional Systems (J & JIS) para obter permissão. Este disse que tinha restrições em permitir que os Drs. Parodi, Veith e Marin usassem o stent, temendo que isso pudesse prejudicar a avaliação da FDA dos stents coronários Palmaz-Schatz que estavam em processo final de aprovação^2^.

Veith, então, solicitou uma reunião com Marvin Woodall, presidente da J & JIS, para verificar se ele poderia ser persuadido a permitir o procedimento baseado em considerações compassivas. Uma reunião entre Marshall, Woodall, Veith e Marin ocorreu em um restaurante no Marriott Hotel adjacente ao aeroporto de Newark. Após uma conversa de 4 horas e demonstrações de preocupações com o bem-estar do paciente se não fosse tratado, os funcionários da J & JIS cederam e permitiram^2^.

Quando se fala em hospitais nos Estados Unidos, pensa-se em instalações modernas, com diversos equipamentos disponíveis. Entretanto, o Montefiore, embora vinculado ao *Albert Einstein College of Medicine*, se localiza no Bronx, uma região pobre de Nova York. Dessa forma, as instalações desse hospital à época não eram das mais modernas e bem-equipadas. Houve dúvida por parte da equipe argentina quanto à viabilidade do procedimento naquelas condições.

No entanto, o procedimento foi realizado sob anestesia local em 23 de novembro de 1992. Os Drs. Juan Parodi e Carlos Schonholz, junto com os Drs. Marin, Veith e Cynamon, realizaram o procedimento por meio de uma arteriotomia femoral direita^2^. Usando um antigo equipamento de fluoroscopia digital, o enxerto foi posicionado e liberado na aorta^2^.

A recuperação foi favoravelmente diferente da de uma cirurgia convencional^2^. A tomografia computadorizada pós-procedimento e ultrassonografia dúplex atestaram a exclusão do aneurisma. O paciente teve alta alguns dias depois e permaneceu livre de sintomas até sucumbir às comorbidades cardiopulmonares, aproximadamente nove meses após o procedimento^2^.

O Dr. Jonh J. Bergan escreveu muitos anos antes dessa técnica sobrepujar a cirurgia convencional: “Em cirurgia vascular, não ocorreu nenhuma mudança para melhor sem a qual homens sábios e bons não se opuseram. Essa mudança é inevitável”^2^. Após a realização desse procedimento nos Estados Unidos, a técnica se difundiu. O Dr. Parodi e sua equipe foram convidados a viajar o mundo inteiro para ensinar e demonstrar essa enorme mudança de paradigma no universo vascular. O reticente *Vascular Establishment* cedeu. Uma nova era foi inaugurada. O universo vascular nunca mais foi o mesmo.
